# Cerebellar Micro Complex Model Using Histologic Boolean Mapping Simulates Adaptive Motor Control

**DOI:** 10.1007/s12021-025-09730-9

**Published:** 2025-06-17

**Authors:** Gregoris A. Orphanides, Christoforos Demosthenous, Ariadni Georgianakis, Vasilis Stylianides, Konstantinos Antoniou, Petros Kyriacou, Andreas A. Ioannides, Alberto Capurro

**Affiliations:** 1 Queen Mary University of London, 4 Newark Street, Mile End Road, London, E1 4 NS UK; 2 AAI Scientific Cultural Services Ltd, 1065 Nicosia, Cyprus

**Keywords:** Intention tremor, Histologic Boolean Mapping, Cerebellar model, Motor control, Boolean algebra, Variable Neuronal Response

## Abstract

Despite extensive cerebellar research, the functional role of individual cerebellar micro complexes (CmCs) in motor coordination remains debated. This study aimed to utilise a reductionist approach to model the CmC function in motor control using the Histologic Boolean Mapping (HBM-VNR) framework and validate it through replication of features observed in the literature. HBM-VNR modelled each neuron within the CmC as a Boolean expression derived from its architectural connectivity. The model incorporates the Variable Neuronal Response (VNR) synaptic model, introducing probabilistic post-synaptic firing to reflect physiological variability. Motor control dynamics follow the cerebellar brain inhibition phenomenon, where Deep Cerebellar Nucleus (DCN) firing activates the antagonist muscles. The model performed the task of feedback-control in an idealised joint following a desired sinusoidal position. HBM-VNR produced a minimalistic model that reproduced adaptive compensation to external forces and predicted intention tremor when CmC population was reduced, and the expected ethanol induced motor impairments. Simulated firing patterns of the DCN and Purkinje cell showed patterns resembling real recordings both in physiological and pathological situations. The Shifting Central Frequency Hypothesis (SCFH) was suggested to explain the CmC comparator functionality. This study presents HBM-VNR as a histologically grounded modelling approach for neural circuits. HBM-VNR simulated adaptive motor control and predicted neocerebellar syndrome symptomatology and alcohol intoxication effects. SCFH offers a computational mechanism consistent with the cerebellar internal model theories and places CmC as the basis for motor learning in line with the literature, positioning HBM-VNR as a scalable framework for neuroanatomical modelling.

## Introduction

The cerebellar circuitry is formed by many functional units, named cerebellar micro complexes (CmCs) (De Benedictis et al., 2022). Each of these units consists of a mossy fiber afferent that projects directly to the deep cerebellar nuclei (DCN) and to several granule cells. The parallel fibers emerging from the granule cells establish synaptic connections with the Purkinje cells which subsequently project back to the DCN. In contrast, climbing fiber afferents directly innervate both the Purkinje cells and the DCN (Chaumont et al., 2013). A complex variety of interneurons, mainly GABAergic, provide feedback to the granular cells and Purkinje neurons (Zhou et al., 2020). These micro complexes are also modulated by monoaminergic and cholinergic inputs, which set the activity level of the network (Schweighofer et al., 2004). The basic function of this network would be to provide a correction signal to match the actual position of the eyes and limbs with a planned motor program. The function of the error signal is to achieve a match for effective motor control (Popa et al., 2016). In very simple terms, the information about the actual position of the body arrives through the mossy fibers (Tuthill & Azim, 2018), while the input carrying the intended settings is provided by the climbing fibers. The correction signal is gathered at the level of the DCN and is thought to have inhibitory effects acting by decreasing tonic excitation in two main pathways: the superior cerebellar peduncles to the motor cortex (via the thalamus), and the inferior cerebellar peduncles to the spinal cord through the vestibular and reticulate nuclei (De Benedictis et al., 2022).

Functions controlled by the cerebellum include monitoring and correcting muscle movements, maintaining balance via communication with the vestibular system and sending synchronisation signals to the cortex (Morton & Bastian, 2004). Nevertheless, the cerebellum is unique as it consists of a crystalline structure made from repeated CmCs. The histological structure of the cerebellum was first described by Cajal during the early twentieth century (Ramón y Cajal, 1909) and was later electro-physiologically confirmed by (Eccles et al., 1967), yet there is ongoing uncertainty about its precise role. Later studies have provided insights into the interactions between the neurons of the CmC and how they can result in specific long-term synaptic plasticity, i.e., long-term depression and long-term potentiation. Despite this, the mechanism of how repeating identical circuits result in the observed versatility of the cerebellum remains an open question. This study modelled each CmC separately and then combined multiple parallel units, providing an approach to explain the observed dichotomy between structural simplicity and functional complexity.

Detailed neuronal models usually involve the release of neurotransmitters on the synaptic cleft from the pre-synaptic membrane, which can induce an excitatory post-synaptic potential (EPSP) or inhibitory post-synaptic potential (IPSP) depending on the nature of the neurotransmitter (Purves et al., 2001). If the firing threshold is reached, a spike is produced in the post-synaptic neuron. This widely accepted model is supported by single neuron recordings (Coombs et al., 1955a, b; Eccles & Rall, 1951) and also explains temporal and spatial summation. However, a limitation of this model is the assumption that the release of neurotransmitters always causes the same response in the post-synaptic membrane, which cannot account for the variable response observed in neuronal circuit recordings (Scaglione et al., 2011). For example, identical visual stimuli can cause different responses in the same neuronal circuit (Kenet et al., 2003). By averaging multiple single trials (STs) in magnetoencephalography (MEG) and electroencephalography (EEG) studies, the common component can be preserved (often low-frequency component) whilst removing the randomness of each trial (high-frequency component). Working memory studies provide another example of this response variability (Nakuci et al., 2023). In recent years this “randomness” has been theorised to constitute an important role in the generation of complex human behaviour and emotions (Waschke et al., 2021).

This computational study introduces a new synapse model, the “Variable Neuronal Response” (VNR) model which accounts for a degree of chance within the post-synaptic response. VNR is based on the molecular dynamics of neurotransmitter-receptor interactions. Every time the pre-synaptic membrane depolarises in response to an action potential, a fixed amount of neurotransmitter is released onto the synaptic cleft, with a pre-defined half-life due to its removal and re-uptake by the pre-synaptic membrane and glial cells. Concurrently, when a singular neurotransmitter binds with a receptor, a fixed response occurs in the post-synaptic membrane; however, the number of successful bindings before removal is not predetermined. Additionally, VNR introduces the concept that a perceived collective frequency from multiple neurons can be individually modelled as a firing probability. Where a neurotransmitter release temporarily alters the neurons firing probability whereas neuromodulators exert a longer lasting change in the neurons firing probability. The VNR synaptic model is supported by Maass, (1997) who demonstrated that rate-coded systems can exhibit complex functions without relying on precise spike timing. Additionally, VNR is supported by Faisal et al. (2008) who showed that biological neurons operate under significant intrinsic noise that contributes to flexible motor control. Through the VNR synaptic model this study introduces the Histologic Boolean Mapping (HBM) method to translate neural histologic maps to Boolean algebra expressions and collectively the HBM-VNR framework.

Cerebellum models span a wide range of scales, ranging from single-neuron models to large-scale circuit simulations. At the single neuron scale, Hodgkin-Huxley-type models simulate the time evolution of a neuron's membrane potential using coupled differential equations formed by the sum of the capacitive current (passive membrane properties), the leak current and the voltage dependent ionic currents (Hodgkin & Huxley, 1952). The Ionic model was later reduced, while still reproducing the same behaviour with less equations such as in the case of the Fitzhugh Nagumo model (FitzHugh, 1961; Nagumo et al., 1962). The leaky integrator model was also introduced simulating only the passive properties of the membrane (RC circuit), artificially annotating a spike when the voltage crosses a threshold, followed by a reset of the voltage to a resting value, where it is clamped during a refractory period (Gerstner & Kistler, 2002). These models often utilise alpha functions to simulate post-synaptic potentials (Rall, 1967). Recent cerebellar modelling efforts have leveraged these principles with Masoli and D’Angelo, (2017) developing a high-fidelity Purkinje model that simulates the detailed membrane properties of Purkinje cells using multi-compartment Hodgkin-Huxley-type frameworks. On the other hand, Geminiani et al. (2024) focused on reconstructing the dynamics of the cerebellar micro circuitry utilising cell type specific characteristics whereas Lorenzi et al. (2023) introduced mesoscale population-level cerebellar models. In comparison, the HBM-VNR framework employs a binary, probabilistic approach grounded in histological connectivity. Each neuron is modelled as a Boolean unit that receives afferent input and produces a firing state (0 or 1) output for each time sample. This state is dependent on its synapse specific parameters but also on the randomisation factor introduced by the VNR model. Thus, the HBM-VNR approach aims to simulate the complexity of neural circuit dynamics without specifically simulating each neuron’s membrane potential.

This article presents, a case study, where the HBM-VNR approach was used to simulate a motor control system utilising the cerebellar micro complex architecture with a reductionist approach. The neural network model consists of elemental random neurons and synapses subjected to plausible plasticity rules. The model aims to achieve “position following” functionality and check its validity by predicting the known deficits of cerebellar dysfunction (Schmahmann, 2004). To assess the model's functionality, it was tasked to provide feedback-control to the motor signal applied to an idealised joint that followed a desired sinusoidal position. External forces were also implemented to further validate the capabilities of the model. HBM-VNR includes the main accepted facts about neural interactions in the different synapses of the circuit and can simulate the matching between intended and actual muscular tone. Intention tremor like behaviour was observed by decreasing the number of functioning units (neocerebellar syndrome) whereas intention tremor and dysmetria were observed when simulating the synaptic modifications due to ethanol in acute alcohol intoxication. Additionally, this study proposes the Shifting Central Frequency Hypothesis (SCFH) to explain the comparator functionality of the cerebellum that aligns with the internal model theories (D’Angelo & Casali, 2013; Ito, 2006) while also providing a computational mechanism based on the cerebellum histology. Furthermore, this study through the HBM-VNR approach provides a framework for inferring the function of a neural circuit based on its architecture.

## Methods

### CmC Model Implementation Using Histologic Boolean Mapping

The HBM-VNR approach requires a non-deterministic synapse model as its basis. Thus, it utilised the VNR synapse model as part of its methodology which allows for post-synaptic response variability. To introduce variability, a random number generator following a uniform distribution was used (Eq. 1). Increasing the number of receptors on the post-synaptic membrane enhances the probability and number of interactions that can occur and vice versa. This was coded as a Synaptic Strength (SyS) variable (Eq. 2). SyS can also account for multiple neurons of the same type feeding on the same post-synaptic membrane, while a negative multiplier is used for inhibitory neurotransmitters. Finally, if the summed action of excitatory and inhibitory neurons (spatial summation) is enough to reach the Depolarization Threshold (DT), an action potential is generated on the post-synaptic membrane. DT was set to 0.15 to reflect the normalised difference between the resting potential and spike threshold, expressed as a fraction of the difference between the resting potential and the spike peak potential. The value of DT can be independently adjusted to replicate the effect of neuromodulators. To account for the effects of neuromodulators which exert long-term effects on the excitability of neurons (Bazzari & Parri, 2019), DT is also considered as a variable in the model. This model aimed to reproduce and incorporate each anatomical synapse present on the CmC using a set of Boolean algebra expressions and implemented the simulation using MATLAB 2022b (The MathWorks Inc., 2022).

Each CmC synapse was modelled separately using a unique equation (Eqs. 5–10). A simplified diagram can be seen in the Fig. 1 of Kano and Watanabe (2017). These set of equations were numerically simulated in the sequence presented below for each time sample. Hence, within a single sample the input is received from the afferents (climbing and mossy fibers), and an output is sent to the DCN. As the model does not account for temporal summation, each time sample is independent, except the synaptic modifications carried to the following time samples. In electrophysiological recordings the activity of neurons is measured by their frequency (Hz) which is defined as the number of spikes within 1 s. This model translated spike frequency to firing probability which is defined as the probability that the neuron fires within each time sample. Equation 4 describes the relationship between firing probability and spike frequency for a given sampling frequency. Each neuron is modelled by a Boolean variable meaning that it can either have the value of 0 or 1, indicating a resting state or depolarised state respectively. For the afferents, a different modelling rationale was used where their state is determined from the firing probability (Eq. 5). The following approach was selected accounting to the fact that the previous circuitry feeding into the afferents was not modelled explicitly. However, the input frequencies can be assumed to depend on the position of the joints (mossy fibers) and on the motor signal (climbing fibers). The neurons following the afferents were calculated with their unique Boolean algebra equations (Eqs. 6 – 10). Notably, the SyS and DT are independent and can be different for each neuron. X is defined as a random variable from a uniform distribution between 0 and 1.

1 $$\text{X }=\text{ U }(\text{0,1})$$

2 $$\text{Synapse Strength }(\text{SyS}) = [0,\text{ inf}]$$

3 $$\text{Depolarisation Threshold }(\text{DT}) = [0,\text{ inf}]$$

4 $$\text{Firing probability}= \frac{\text{Spike Frequency}}{\text{Model Sampling Frequency}}$$

5 $$\text{Afferent }=\text{ X }\ge (1-\text{ Firing Probability})$$

6 $${\text{Granule cell}}_{\text{n},\text{t}} = (\left(\text{X}* {\text{SyS }}_{\text{mf}-\text{granule},\text{n }}*1\left({\text{Mossy fibre}}_{\text{n},\text{t}}\right)\right)- (\text{X }* {\text{SyS }}_{\text{golgi}-\text{granule},\text{n}}* 1({\text{Golgi cell}}_{\text{n},\text{t}-1})))> {\text{DT}}_{\text{granule},\text{n}}$$

7 $${\text{Golgi cell}}_{\text{n},\text{t}} = (\text{X }* {\text{SyS }}_{\text{granule}-\text{golgi},\text{n}}* 1({\text{Granule cell}}_{\text{n},\text{t}}))> {\text{DT}}_{\text{granule},\text{n}}$$

8 $$\text{I}{\text{nhibitory interneuron}}_{\text{n},\text{t}} = (\text{X }* {\text{SyS }}_{\text{granule}-\text{ii},\text{n}}* 1({\text{Granule cell}}_{\text{n},\text{t}}))> {\text{DT}}_{\text{ii},\text{n}}$$

9 $${\text{Purkinje cell }}_{\text{n},\text{t}}= (\left(\text{X }* {\text{SyS }}_{\text{granule}-\text{purkinje},\text{n}} * 1\left(\text{G}{\text{ranule Cell}}_{\text{n},\text{t}}\right)\right)+ \left(\text{X }* {\text{SyS }}_{\text{cf}-\text{purkinje},\text{n}}* 1\left(\text{C}{\text{limbing fibre}}_{\text{n},\text{t}}\right)\right)- (\text{X }* {\text{SyS }}_{\text{ii}-\text{purkinje},\text{n}}* 1({\text{Inhibitory interneuron}}_{\text{n},\text{t}})))> {\text{DT}}_{\text{purkinje},\text{n}}$$

10 $${\text{DCN }}_{\text{n},\text{t}}= ((\text{X }* {\text{SyS }}_{\text{mf}-\text{dcn},\text{n}}* 1\left({\text{Mossy fibre}}_{\text{n},\text{t}}\right)+ \left(\text{X }* {\text{SyS }}_{\text{cf}-\text{dcn},\text{n}}* 1\left({\text{Climbing fibre}}_{\text{n},\text{t}}\right)\right)- (\text{X }* {\text{SyS }}_{\text{purkinje}-\text{dcn},\text{n}}* 1({\text{Purkinje Cell}}_{\text{n},\text{t}}))> {\text{DT}}_{\text{dcn},\text{n}}$$,

where “t” represents the current time sample of the model and “n” represents the number of CmCs running in parallel.

**Fig. 1:**
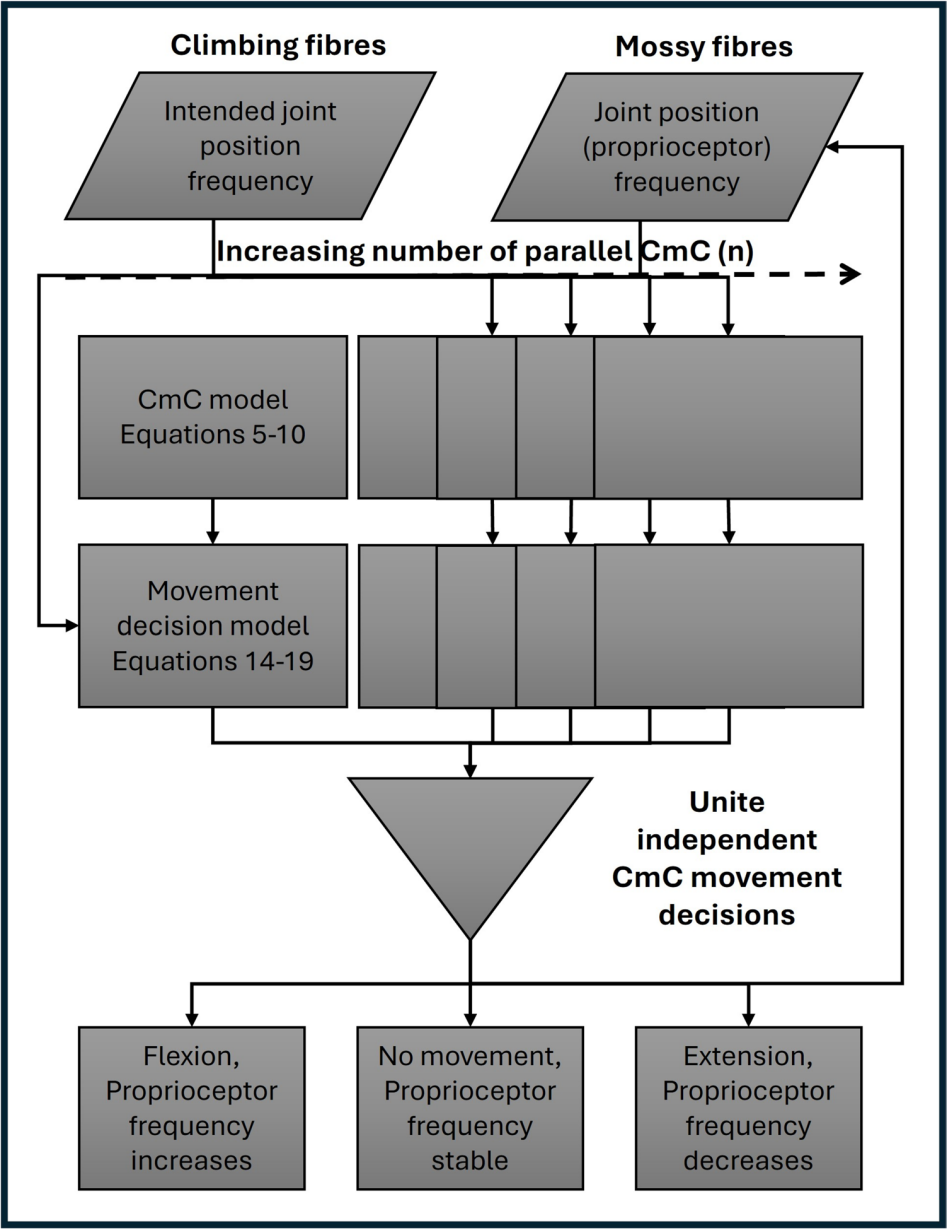
Schematic diagram outlining the steps for the HBM-VNR feedback-loop motor control system

### Relationship Between Firing Probability and Firing Frequency

When measuring the electrical activity of neurons or population of neurons (e.g., extracellular recordings) frequency is often used as a marker of neuronal activity. Frequency is measured in Hertz and corresponds to the number of events such as spiking of neurons that occur in every second. Signal analysis comes with an inherent dichotomy between temporal resolution and spatial resolution (frequency) where to accurately measure the state of one of these components information regarding the other one is lost. This can be exemplified when considering the Fourier transform that provides excellent frequency resolution but at the cost of time resolution. HBM-VNR faces a similar problem when attempting to replicate frequency in a time specific model. To address this issue, HBM-VNR utilised the firing probability instead of the frequency as an indicator of neuronal activity. Equation 4 enables both the conversion of an afferent frequency to firing probability and the conversion of efferent firing probability to frequency. This conversion can be achieved if the sampling frequency (time-step) is known. Let us consider the modelling of a neuron with a firing frequency of 50 Hz. If the sampling frequency is 100 Hz i.e., there are 100 samples of the simulation in each second, then in each individual sample the neuron has a 50% chance of firing. Conversely, if the sampling frequency is doubled, the neuron now has a 25% chance of firing in each time-step, maintaining an average of 50 firings in each second of the simulation. As HBM-VNR does not currently accommodate for temporal summation, the highest sampling frequency that can be used is 250 Hz. This is due to the assumption of independence between time-samples implemented in the HBM-VNR model. Therefore, by selecting an adequate time-step (4 ms), all neurons will return to their resting state by the next time-sample of the simulation, preserving the principle of independence between time-samples.

### Intention Tremor

Intention tremor is calculated by determining the variance between the distance of the mean and actual muscle position (Eq. 12). In Figs. 5 and 6 a moving variance with a window of 5-time samples (half window = 2-time samples) is used to measure tremor across time.

11 $${Mean\;muscle\;position}_{t}= \frac{\sum_{i= t- half\;window}^{t + half\;window}{(muscle\;position}_{i})}{window}$$

12 $${Intetion\;tremor}_{t}= \frac{\sum_{i=t-half\;window}^{t+half\;window}{(\left|{muscle position}_{i}- {Mean\;muscle\;position}_{i}\right|)}^{2}}{window}$$,

where “t” represents the current time sample of the model and “n” represents the number of CmCs running in parallel.

### Muscle Movement Model Rationale

In this study, the muscle control system was inspired by the Cerebellar Brain Inhibition (CBI) theory supported by Transcranial Magnetic Stimulation experiments. CBI is a phenomenon where the excitation of the primary motor cortex (M1) followed by the excitation of the cerebellum reduces the amplitude of the muscle evoked potential (Tanaka et al., 2018). Thus, excitation of the Purkinje cells results in the tonic inhibition of the DCN to M1 pathway. To articulate in another way, an excitation of the Purkinje cells results in M1 inhibition. Concurrently, CBI is targeted towards the antagonist muscles, which paradoxically results in assisting the planned movement (Panyakaew et al., 2016). CBI assists the desired movement and causes a reduced Muscle Evoked Potential (MEP). The reason underlying this apparent paradox is that the tonic inhibition from the CBI reduces the activity of the antagonist muscles to a larger extent than the agonists. To replicate the described behaviour in the model, for each CmC a comparison between the DCN and motor signal was performed to determine muscle power. If only the DCN fires, then muscle position shifts away from the intended movement (antagonist is active), whereas if only the motor signal is active the muscle position follows the intended plan (antagonist inhibited). A combined firing of DCN and motor signal results in no joint position change. The normalised net result of the interaction between the cortex and DCN is then multiplied by the muscle strength to account for the muscle power (Eqs. 13). The described logic was implemented according to the HBM-VNR method using Eq. 14–19 inspired from the motor cortex and cerebellar control via the superior peduncle. The advantage of these equations is that they allow independent comparison for each CmC and additionally introduces a constraint to prevent the same circuit causing activation of both the agonist and antagonist muscles. A constant force on the joint has also been implemented. This force is simply added or subtracted to the muscle power at each time-sample (Eq. 20). The calculated muscle power is added to the joints position on the current time-sample to calculate its position in the next sample (Eq. 21). Movement error is simply defined as the absolute value of the difference between the desired and actual joint position (Eq. 22).

13 $${Muscle\;Power}_{t}=(\frac{{\sum }_{i=1}^{n}\left({{Motor\;signal}_{i,t}- DCN}_{i,t}\right)}{n} * Muscle\;strength)$$

14 $${P1}_{n,t}=\left(X * {SyS}_{DCN-P1,n}* 1\left({DCN}_{n,t}\right) - X * {SyS}_{motor-P1,n}* 1({Motor\;signal}_{n,t})\right)>{DT}_{P1,n}$$

15 $${P2}_{n,t}=\left(X * {SyS}_{motor-P2,n}* 1\left({Motor\;signal}_{n,t}\right) - X * {SyS}_{DCN-P2,n}* 1({DCN}_{n,t})\right)>{DT}_{P2,n}$$

16 $${P3}_{n,t}=\left(X * {SyS}_{P1-P3,n}* 1\left({P1}_{n,t}\right) +X * {SyS}_{P2-P3,n}* 1({P2}_{n,t})\right)>{DT}_{P3,n}$$

17 $${Flexor}_{n,t}=\left(X * {SyS}_{P3-Flexor,n}* 1\left({P3}_{n,t}\right) -X* {SyS}_{DCN-Flexor,n}* 1({DCN}_{n,t})\right) * Flexor\;Excitability>{DT}_{Flexor,n}$$

18 $${Extensor\_interneuron}_{n,t}=\left(X * {SyS}_{P3-Extensor\_interneuron,n}* 1\left({P3}_{n,t}\right) - X* {SyS}_{DCN-Extensor\_interneuron,n}*1({DCN}_{n,t})\right)>{DT}_{Extensor\_interneuron,n}$$

19 $${Extensor}_{n,t}=\left(X * {SyS}_{P3-Extensor,n}* 1\left({P3}_{n,t}\right) - X* {SyS}_{Extensor\_interneuron-Extensor,n}* 1(E{xtensor\;interneuron}_{n,t})\right)* Extensor\;Excitability>{DT}_{Extensor,n}$$

20 $${Muscle\;Power}_t=\left(\frac{\sum_{i=1}^n\left({{Flexor}_{i,t}-Extensor}_{i,t}\right)}n\ast Muscle\;strength\right)+Force\;on\;joint$$

21 $${Joint\;position}_{t+1}= {Joint\;position}_{t} + {Muscle\;Power}_{t}$$

22 $${Movement\;error}_{t}= \left|{Actual\;position}_{t}- {Desired\;position}_{t}\right|$$

, where “t” represents the current time sample of the model and “n” represents the number of CmCs running in parallel.

### CmC HBM-VNR Function Summary

HBM-VNR’s application to the cerebellum aims to explore the role of the CmC and not of the cerebellum as a whole in motor movement, as a case study of this methodologies’ validity. A summary pipeline of the model steps are illustrated in Fig. 1. The synaptic connectivity of the model follows the histological descriptions reported in the literature (Ramón y Cajal, 1909; W. Zhang & Linden, 2006). HBM-VNR considers that in every joint its position is encoded using the muscles proprioceptors firing frequency. Each position is encoded by a unique frequency value with a high frequency corresponding to muscle flexion and low frequency to muscle extension. These are then transmitted to the CmC using the mossy fibers that then feed into the granule cells forming the parallel fibers. Similarly, the climbing fibres set the desired joint position by matching their firing frequency to correspond to the proprioceptors firing frequency at the desired position. Therefore, if joint flexion is required the climbing fibres will fire with a high frequency and vice versa. When referring to a joint this study assumes an ideal joint with a flexor and extensor muscle and not to a particular joint within the body.

For each sampling step, the firing probability of the mossy and climbing fibres are determined, (as discussed above) and the same input is fed to all the parallel modelled CmC; each producing a Boolean value of the DCN by running Eqs. 5, 6, 7, 8, 9, 10. Every CmC is independent in the sense that there is no communication between them, except for the summation of the final motor decision. Even though the same input is given to each CmC, their output is not identical both due to the randomisation factors introduced by HBM-VNR and due to potential differences in synaptic plasticities. SyS and DT variables do not have the same values in each circuit as they are altered according to the synaptic plasticity rules shown in Table 2. The HBM-VNR model of the cerebellum assumes that there are no parallel connections between the micro-complexes that control a single joint. However, communications between different joints and somatotopic maps are well documented in the literature and are indispensable for motor control (Eskiizmirliler et al., 2002). To determine the motor output independently for each CmC, the computed DCN value is combined with the input climbing fibre value according to Eqs. 14–19. This gives a binary value to the Flexor (Eq. 17) and Extensor (Eq. 19) Boolean variables. Due to the equations utilised, it is unlikely that the extensor and flexor variables are both set to 1. If the flexor variable is set to 1, then the joint position value increases resulting in an increased proprioceptor frequency, whereas activation of the extensor variable results in decrease in joint position value and proprioceptor frequency. Equation 20 is used to summarize the motor effects of all the independent CmC and Eq. 21 is used to update the joint position. A summary of the abreviations used in this manuscript can be seen in Table 3.

### Tasks and Expected Capabilities

The HBM-VNR model simulates individual CmCs to investigate how their histological architecture contributes to motor control. A reductionist approach was used that focused on the function of the CmC, which does not model the more complicated function and interaction between other components of the motor system. The model incorporates synaptic plasticities that allow the interaction and alterations in firing frequency to be observed in physiology and pathology. More specifically HBM-VNR can model the firing pattern of any neuron within the CmC given a firing pattern from the CmC afferents. Synapse specific changes can be applied by altering the parameters to emulate changes in firing patterns and frequencies observed in each neuron type of the CmC. Additionally, the model allows to perform movement control by enabling HBM-VNR to act as a closed-loop system. Moreover, it facilitates the prediction of effects of pathology such as an imbalance between muscle power and number of CmC and changes in synaptic strengths on movement.

### Temporal Summation Model

The majority of the results presented in this study utilise the HBM-VNR model without utilising temporal summation. However, a model able to perform temporal summation is also showcased in Section "Temporal model of HBM-VNR". In order to simulate temporal summation, the sum of the net effect of pre-synaptic neurotransmitter release is carried to the next time-step through the application of a linear decay transformation (Eq. 24). In case the depolarisation threshold is reached in a time-step, a value of −1 is imposed to avoid another firing of the neuron, until the membrane potential is restored to 0 (representing an absolute refractory period). The refractory period was set to 3 ms which plausible for CmC neurons (Cao et al., 2017; Fernandez et al., 2007). This variable is parameterised; therefore it can be changed in future simulations.

24 $$Sampling\;step= \frac{1000}{Model\;Sampling\;Frequency}$$

25 $$Decay\;factor=\frac{(Refractory\;period-Sampling\;step)}{Refractory\;period}$$

26 $${Neurotransmitter\;effect}_{neuron,n,t+1}=\left(\left(\neg {Neuron\;state}_{neuron,n,t}*{Neurotransmitter\;effect}_{neuron,n,t}\right)+\left(-1\left({Neuron\;state}_{neuron,n,t}\right)\right)\right)*Decay\;factor$$,

where “neuron” represents the specific type neuron within the CmC, “t” represents the current time sample of the model and “n” represents the number of CmCs running in parallel.

### Time–frequency Analysis

Time–frequency analysis was performed with morse wavelets using the “cwt” MATLAB function. A frequency range of 0.5-100 Hz was produced using a time frequency bandwidth value of 120 and gamma set to 3 (Table 1).

**Table 1:** HBM-VNR variable and description appendix

Variable	Description	Typical Value/Range
${SyS }_{x-y,n}$(Synaptic Strength)	Represents the strength of a synapse; affecting probability of post-synaptic firing “x” represents pre-synaptic membrane; “y” represents post-synaptic membrane “n” is the index of the parallel CmC circuit	1.5 Modified according to synaptic plasticity rules seen in Table 2 for Section "CmC adaptive and learning behaviour"
${DT}_{x,n}$(Depolarisation Threshold)	Threshold required for a neuron to fire. Expressed as a fraction of the distance from the resting potential to the action potential peak “x” represents post-synaptic membrane “n” is the index of the parallel CmC circuit	0.15
CmC count	Number of cerebellar micro complexes simulated in parallel	100,000
${Neuron state}_{neuron,n,t}$	Boolean representation of neuronal state (0 = rest, 1 = firing) “neuron” represents post-synaptic membrane “n” is the index of the parallel CmC circuit “t” represents the time-sample index	Binary (0 or 1)
Firing Probability	Model equivalent of firing frequency, dependent on sampling frequency	0–1 (converted to Hz via Eq. 4)
Sampling Frequency	Temporal resolution of the model	250 Hz (non-temporal model) 500 Hz (temporal model)
Sampling Step	Temporal step of the model	4 ms (non-temporal) 2 ms (temporal) (Determined via Eq. 24)
Muscle Strength	Maximum force a modelled muscle can exert in a sample	10
Force on joint	Additive or subtractive value to muscle power (Eq. 20)	Set to 0 (Eq. 20)
Refractory period	Time in ms for neurons to be able to depolarise after activation	3 ms
${Neurotransmitter effect}_{neuron,n,t}$	Net effect of neurotransmitters on post -synaptic membrane, “neuron” represents post-synaptic membrane “n” is the index of the parallel CmC circuit “t” represents the time-sample index	−1—+ 1 (Dependent on neuron specific equation)
Decay factor	Linear parameter determining proportion of neurotransmitter effect carried to the next sample. Dependent on model sampling frequency	2/3 (Determined via Eq. 25)

**Table 2:** Summary of potentiations according to literature

*Type of potentiation* *Long term depression (LTD)/Long term Potentiation (LTP)*	*Condition reported in literature*	*Model Purkinje firing probability*	*Model DCN firing probability*
Parallel fiber—> Purkinje cell LTD (Ito & Kano, 1982; Ohtsuki et al., 2009)	Parallel fiber and Climbing fiber co-activation	Decreased	Increased
Parallel fiber—> Purkinje cell LTP (Wang et al., 2014)	Isolated High-frequency activation of the Parallel fiber	Increased	Decreased
Mossy fiber—> Granule cell LTP (D’Angelo & De Zeeuw, 2009; Pugh & Raman, 2006)	High-Frequency activation of the mossy fiber	Increased	Decreased
Climbing fiber—> Purkinje cell LTP (Hansel & Linden, 2000; Ito, 2006)	High-Frequency activation of the Climbing fiber	Increased	Decreased
Mossy fiber—> DCN LTD (Gao et al., 2012; Hansel et al., 2001)	Low-frequency activation mossy fiber	No effect	Decreased
Purkinje cell—> DCN LTD (W. Zhang & Linden, 2006)	Co-existence of DCN activation and Low-frequency Purkinje cell activation	No effect	Increased

## Results

### Single CmC Observations

#### Single Cerebellar Circuit Across Movement

**Fig. 2:**
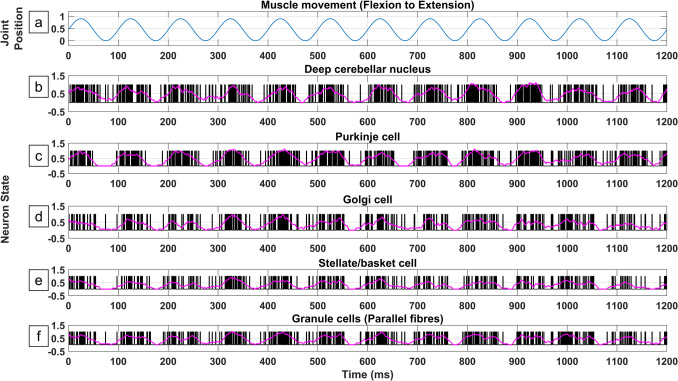
Population firing pattern of the different neuronal components of a single cerebellar circuit while modelling a single joint flexion and extension across time. a) Joint position generated using a sinusoidal function, with 1 being complete flexion and 0 being complete extension. b-f) Raster plot of the cerebellar circuit neuronal types. Value of 0 indicates neuron is in rest, value of 1 indicates neuronal firing. The magenta line represents the neuronal firing rate (moving average). A refractory period of 3 ms was implemented in the neuronal firing

Even though the cerebellar circuitry has been widely discussed within the literature, its exact function remains widely debated, with many models arising to describe the function of both its inputs (afferents) and output (efferent) (Warren & Sawtell, 2016). Figure 2 illustrates the depolarization pattern of the CmC’s neurons under muscle movement (flexion to extension) according to the HBM-VNR implementation (Section "CmC model implementation using histologic Boolean mapping"). A magenta line shows a moving average of the neuron firing rate. In accordance with cerebellar electrophysiological recordings (Heiney et al., 2017; “Neuroscience, 5 th Ed.,” 2012) the frequency of both the DCN and Purkinje neurons show a common increase and decrease in accordance with joint position. Despite this phase dependent pattern, the spike times displayed considerable variability across time. This jitter stems from the randomisation factor included in the model and it is observed in real life recordings (Fig. 5 in” Neuroscience, 5 th Ed.,” 2012). The output frequency of the DCN appears to be correlated with the position of the joint, reflecting the muscle proprioceptor frequency. Therefore, it has a variable pattern dependent on the actual position of the joint and the desired position. In this context, the DCN frequency must be interpreted together with its afferent activity, and not independently. In summary, a fundamental insight from the current analysis is that multiple cerebellar circuits need to be implemented in parallel to generate a stable pattern.

#### Temporal Model of HBM-VNR

**Fig. 3:**
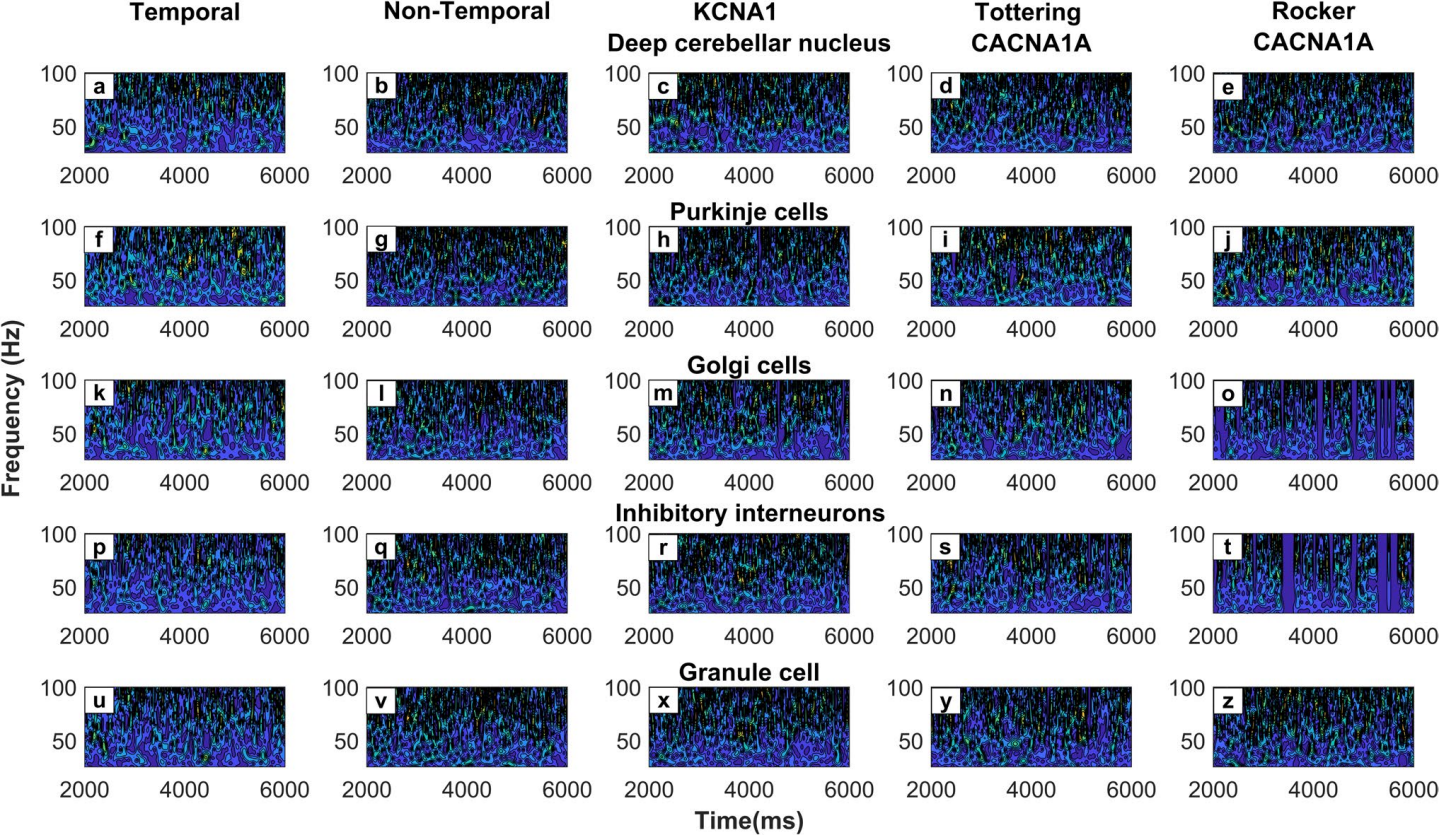
CmC neural component firing frequency spectrum (Section "Time–frequency analysis"). **a**, **f**, **k**, **p**) Firing pattern of temporal model (Section "Temporal summation model"). **b**, **g**, **l**, **q**, **v**) Firing frequency spectrum of non-temporal model. **c**, **h**, **m**, **r**, **x**) Firing frequency when applying KCNA1 mutation effects (Kv1.1 loss of function). **d**, **I**, **n**, **s**, **y**) Firing frequency for tottering CACNA1A mutation (reduction of SyS for affected synapses by 65%). **e**, **j**, **o**, **t**, **z**) Firing frequency for tottering CACNA1A mutation (reduction of SyS for affected synapses by 85%)

Figure 3 illustrates the spectrogram calculated using a morse wavelet time–frequency analysis (Section "Time–frequency analysis") of single CmC neuron types during movement. Each column represents a set of neurons from the same CmC, but representing different conditions indicated by the column title. The first column (Fig. 3a, f, k, p, u) represents the firing frequencies generated by the model that incorporates temporal summation (Section "Temporal summation model") while the second column (Fig. 3b, g, f, q, v) represents the same simulation produced by the standard HBM-VNR model without temporal summation. Similar firing patterns were observed in both simulations. The temporal model spectrograms show a higher resolution in the localisation of spatial activity, which is due to the higher sampling frequency used. The non-temporal model is limited to a 250 Hz resolution to ensure all cells can return to their resting potential in each time-step of the simulation. On the other hand, for the effects of temporal summation to be observed, a higher sampling frequency is required to prevent the membrane potential decaying before the next time-step of the simulation is reached. Given that the scope of this study was to produce a computationally efficient minimalistic model, the decision to use only the non-temporal version was taken. The non-temporal model is more memory efficient as it does not require storing the state of the neuron from previous time-steps. Nevertheless, the use of the temporal model (Fig. 3) would be beneficial to improve the frequency resolutions, or to study more detailed synaptic integration in the context of future studies.

#### KCNA1 Mutation

The KCNA1 gene encodes for the Kv1.1 potassium channel found on the basket cell axon terminals within the CmC and plays an important role in regulating the excitability of the basket cells (Smart et al., 1998). A loss of function mutation in the KCNA1 gene results in basket cell hyperexcitability, leading to increased GABAergic inhibition of the Purkinje cells and subsequent disinhibition of the DCN cells. This is behaviourally expressed as motor dysfunction and as episodic ataxia type 1 (EA1) (Herson et al., 2003). This mutation can be emulated using the HBM-VNR model by decreasing the DT variable of the inhibitory interneurons (Eq. 8). Results are seen in the 3rd column of Fig. 3 (Fig. 3c, h, m, r, x). Implementing this change results in increased activation of the inhibitory interneurons when compared with the baseline condition (Fig. 3l, q). Furthermore, there is a closer resemblance between the activity of granule cells and inhibitory interneurons, indicating a tighter transfer of information between them, as would be expected with a lowered depolarization threshold. Importantly, a subsequent decrease in Purkinje cell activity is seen in Fig. 3h, accompanied by a downward shift in firing frequency compared with baseline. Figure 3g shows a shift from 90 Hz bursts to 70 Hz bursts, with intermittent pauses at 2450 ms and 4250 ms. In addition to this lower firing frequency, there is a notable loss of spike regularity, consistent with experimental observations in Kv1.1-null mice (Martin & Kullmann, 2023; C. L. Zhang et al., 1999). Therefore, the simulated effect of the Kv1.1 mutation in Purkinje cells qualitatively emulates the firing patterns and firing frequency changes reported in the literature (Herson et al., 2003; Raman & Bean, 1997). Furthermore, downstream effects are also observed in the DCN, where episodic overactivity emerges as high-frequency and low-frequency bursts (Fig. 3c), likely driven by the loss of tonic Purkinje inhibition, as reported in the literature (Person & Raman, 2012). These changes reflect the DCN dysregulation associated with EA1 and help explain the episodic motor symptoms seen in both human patients and Kv1.1 mutant mice (Brunetti et al., 2012; Maylie et al., 2002).

#### CACNA1A Rocker and Tottering Mutations

The CACNA1A gene encodes for the α1 subunit of the CaV2.1 voltage gated calcium channel that is highly expressed in the dendrite and axon terminals of Purkinje cells, climbing fibres and presynaptic terminals of the granule cells (Pietrobon, 2010; Zhuchenko et al., 1997). CACNA1A normally plays an important role in allowing the influx of Ca2+ + in the presynaptic membranes to facilitate the fusion of vesicles, thus resulting in the release of neurotransmitters (Indriati et al., 2013). These effects were implemented into the HBM-VNR model by altering the value of their respective SyS variables corresponding to the synapse strength of climbing fibres, granule fibres and Purkinje cells. This study explored the effect of two CACNA1A mutation variants; the mild tottering mutation where the release of neurotransmitters is reduced by 65% and the more severe rocker mutation where SyS is reduced by 85% (Fletcher et al., 2001). In vivo recordings in these mutant mice have shown that both tottering and rocker strains exhibit irregular bursting patterns in Purkinje cell firing, attributed to both intrinsic Purkinje cell dysfunction and impaired excitatory synaptic drive (Cook et al., 2021; Stahl & Thumser, 2014). This phenomenon is observed, in the form of a concordance between low and high frequency bursts in the Purkinje cells. Figure 3i, j reveal activity occurring closely in the 80-100 Hz and 60-80 Hz bands followed by a pause which is not seen in the baseline (Fig. 3g). Additionally, Fig. 3e shows erratic firing activity in the DCN, as the intra-burst frequency is widespread from 40-100 Hz, in comparison to Fig. 3b (non-temporal model) where the intra-burst frequency is concentrated within the 80-100 Hz band, with some activity in the 60 Hz band. These fluctuations are consistent with the loss of regular Purkinje inhibition, as previously described in CACNA1A mouse models (H. Zhou et al., 2014). Figure 3e, f further reveal a marked reduction in Golgi and molecular layer interneuron activity, including intermittent silencing, consistent with reduced glutamatergic drive from granule cells, in the context of impaired presynaptic CaV2.1 function (Indriati et al., 2013). In summary, the burst pause behaviour of the Purkinje cells results in irregular signalling transmitted to the DCN. During bursts of Purkinje cells, rapid inhibition occurs followed by sudden disinhibition pauses of the DCN. This disorganised activity, accumulated from multiple CmC, could result in the observed motor phenotype of ataxia and dysmetria in tottering and rocker mice.

### Population of CmC Behaviour

#### Shifting Central Frequency Hypothesis (SCFH)

According to the wider literature and the accepted histological organization of the cerebellar circuit there are two main inputs; the climbing fibers and the mossy fibers (Wilson et al., 2019). It is generally accepted that mossy fibers carry information from the muscle proprioceptors and therefore carry information regarding the current muscle position (Jörntell, 2016). Nevertheless, there is a debate on the exact function of the climbing fibers, with some studies suggesting it represents the desired position coming from the cortex (Herzfeld et al., 2020) and others considering it to be a “training signal” (Ohmae & Medina, 2015) or error signal (Zang & De Schutter, 2019). At this stage we do not aim to explain the role of each of the cerebellar circuits inputs but rather to understand how they affect the cerebellar circuit output (DCN). To overcome the randomness introduced by the HBM-VNR model, multiple cerebellar micro complexes were simulated simultaneously, and the result was averaged to obtain a mean firing frequency of the entire DCN neuronal population, according to the climbing fiber and mossy fiber firing frequencies. The conversion of the firing probability to the more often used spike frequency (Hz) is derived according to Eq. 4. The SCFH aims to explain the variable output frequency of the cerebellum by suggesting that the climbing fibers set the central (default) output frequency in accordance with the CmC’s synapse modification. The central frequency is then altered depending on the relative frequencies of the mossy and climbing fibers. If the relative frequency of the mossy fiber is less than the climbing fiber, the DCN frequency is reduced. Conversely, if the mossy fiber frequency is greater, the DCN frequency increases. Therefore, if the mossy fiber frequency matches the climbing fiber frequency the DCN frequency is equal to the central (default) frequency.

#### Synaptic Weights Effect On DCN Central Frequency

**Fig. 4:**
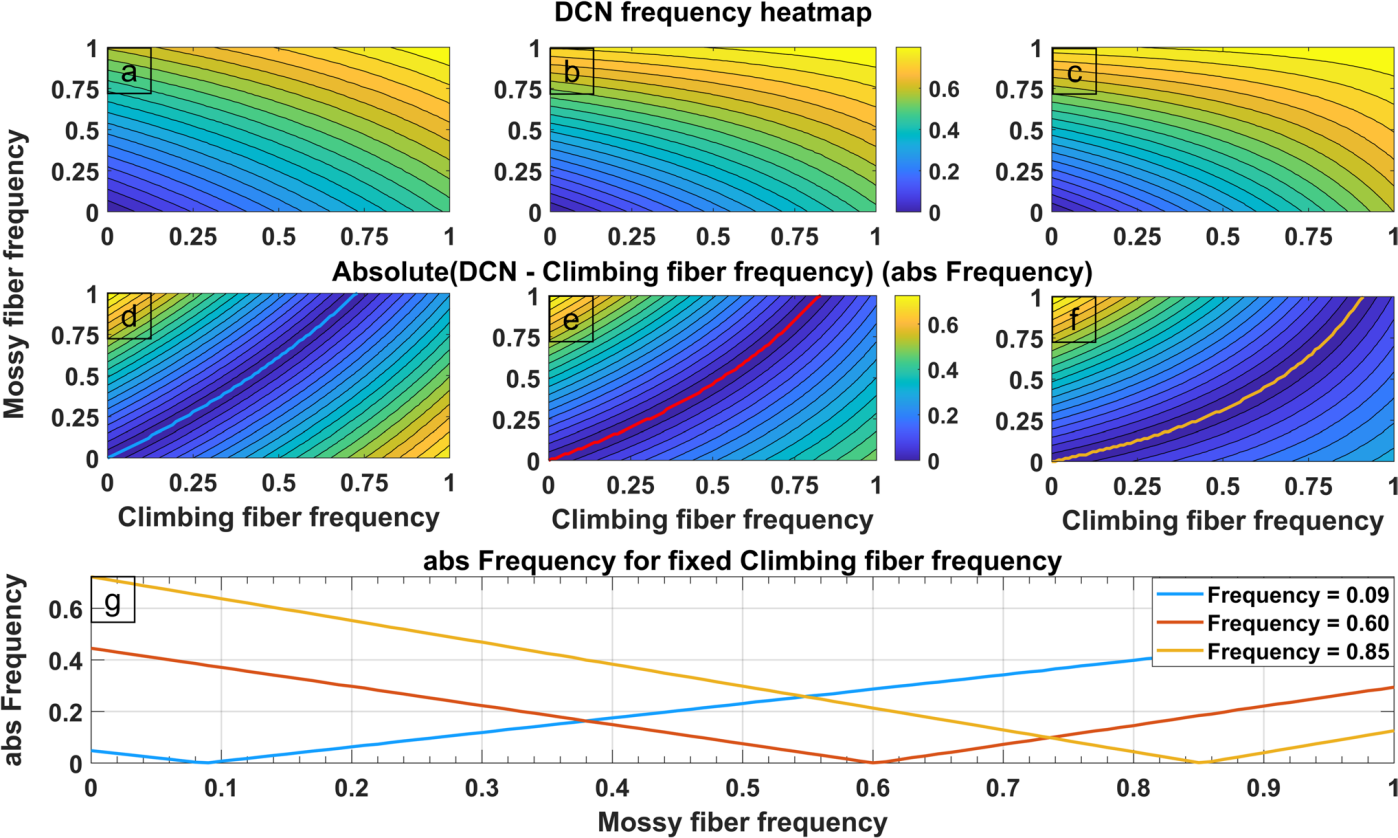
Single deep cerebellar nucleus (DCN) frequency pattern across varying climbing fiber and mossy fiber input frequencies (10^6 modelled CmCs). Frequency is expressed in the model equivalent units of firing probability (Section "CmC model implementation using histologic Boolean mapping", Eq. 4). **a**-**c**) Contour heatmaps of DCN output frequencies. **d**-**f**) Heatmaps of the absolute values of the differences between the DCN and climbing fiber frequencies. The line of symmetry is indicated with a superimposed colour coded curve. The same colour code is used in panel g. **g**) Absolute value of the difference between the DCN and the climbing fiber frequencies, plotted against the mossy fiber frequency for selected constant values of the climbing fiber frequency (indicated in the figure label). The rationale of the selection was the point where the absolute value of the above-mentioned difference reached a minimum

Figure 4 illustrates the basis for the aforementioned SCFH mechanism. Each column represents the same CmC but with altered SyS constants for the Purkinje and DCN cells. The first row shows plots with the climbing fiber and mossy fiber depolarization probability (frequency equivalent) on the y and x axis respectively (Fig. 4a-c). DCN frequency is represented according to the figures color scale. As both mossy fiber and climbing fiber frequency increases, the DCN frequency also increases. To better understand SCFH we subtracted the climbing fiber frequency from the DCN firing frequency and then calculated the absolute value which is displayed on the 2nd row (Fig. 4d-f). A line is further plotted to show the central frequency where DCN matches the frequency of the CF, i.e., the result of the above equation is 0. The plotted line is seen as a line of symmetry indicating that the correction of the cerebellum is not only centred around the CF dependent central frequency, but it is also symmetric (Fig. 4d-g). Changing the CmC’s synaptic weights then shifts the central frequency line. These results suggest that the shift of the central frequency might be associated with synaptic plasticity known to underlie the learning process.

### CmC HBM-VNR Movement Simulations

#### Neocerebellar Syndrome

**Fig. 5:**
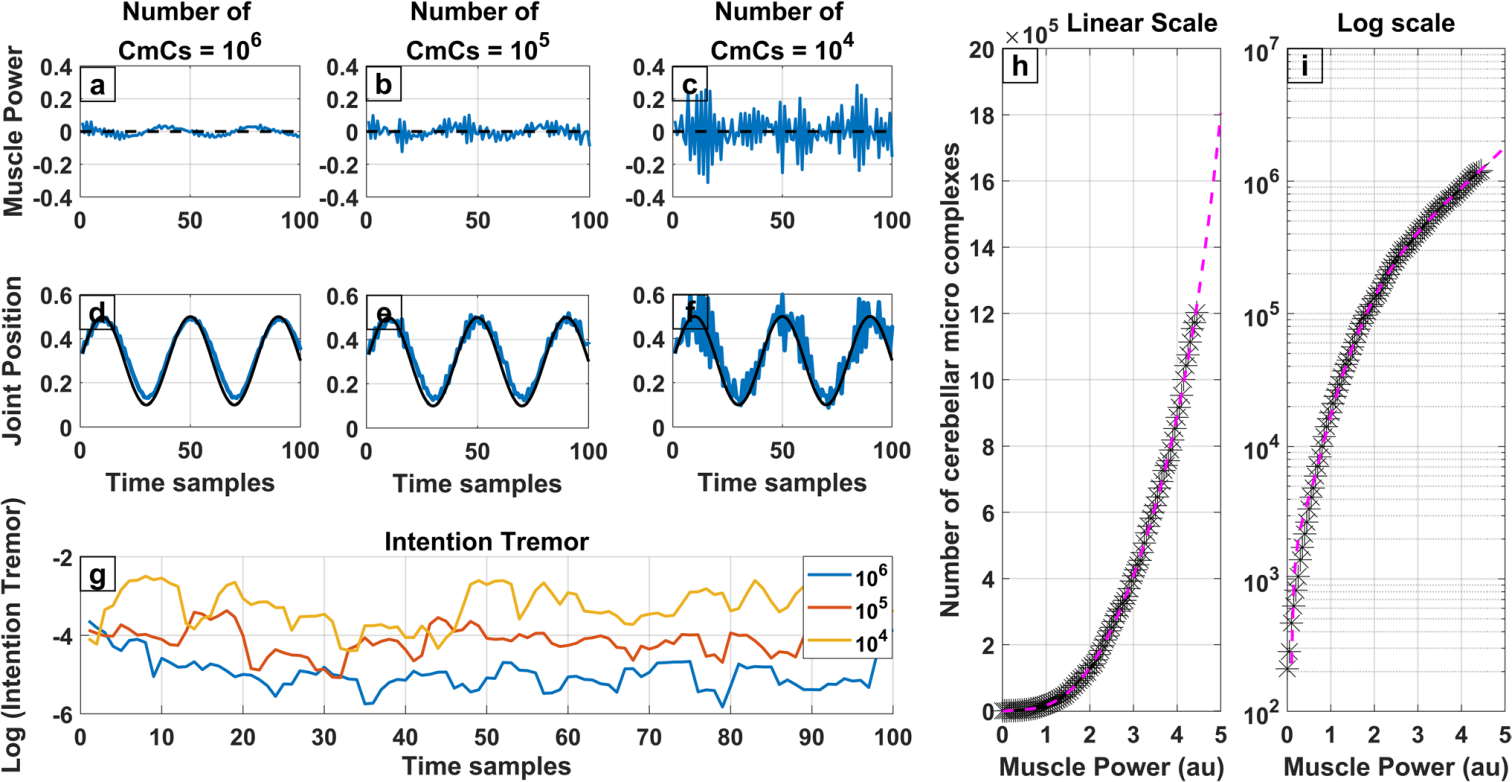
Simulated joint movement while pursuing a desired position and required number of CmCs needed to produce smooth movement. **a**-**c**) The blue line shows muscle power across time. Dashed black line indicates zero power. **d**-**f**) The blue line indicates joint position over time. The black line indicates the desired joint position. **g**) Log of intention tremor (Section "Intention tremor", Eq. 12) over time for different number of parallel CmCs simulated. **h**-**i**) Number of CmCs required to execute movements with very low intention tremor plotted against muscle power (linear scale in panel h and log scale in panel i)

Neocerebellar syndrome is a disorder that results from damage to the cerebellar hemispheres, leading to cell death and to a decrease to the number of functioning cerebellar micro complexes (Manto, 2009). It is most commonly caused by stroke in people aged over 45 and due to genetic causes in the younger population (Joshua et al., 2024). One of the main characteristics of neocerebellar syndrome is the presence of intention tremor, i.e., tremor that co-emerges with voluntary movement (Paredes-Acuna et al., 2024). Differentiating the type of tremor can often act as a differential for possible neurodegenerative diseases, since resting tremor is characteristically seen in conditions affecting the basal ganglia such as Parkinson’s disease (Helmich et al., 2012) in contrast to intention tremor seen in cerebellar dysfunction. Figure 5 illustrates how intention tremor can be triggered due to a reduction in the number of cerebellar micro complexes. A blue line shows the modelled joint position while a black line illustrates the desired joint position (Fig. 5d-f). Moving along the figure columns from left to right the number of cerebellar circuits decreases by a factor of 10 each time. In all columns the joint position tends to follow the desired position (close association between the blue and black lines). However, in the right column where the least number of circuits were used (Fig. 5c, f), the achieved joint position is the least accurate with large over and undershooting which are exacerbated every time the number of circuits decreases. The described movement can be interpreted as intention tremor. Figure 5a-c shows the muscle power which also presents larger variability and a discontinuity when an insufficient number of cerebellar circuits were used. The bottom row conveys the intention tremor calculated according to Section "Intention tremor"using a logarithmic scale to highlight the difference in magnitude between the degrees of intention tremor measured. Hence, the HBM-VNR model predicts the emergence of intention tremor as cerebellar circuits are lost, similarly to the real-life symptoms of neocerebellar syndrome.

#### Relationship of Intention Tremor and Number Cerebellar Circuits

As described above, intention tremor like behaviour, arises when the number of CmCs assigned to a muscle in the model decreases while keeping constant the strength of the muscle. Nevertheless, a relationship between the number of CmCs required to achieve smooth movement and muscle strength also exists. This relationship is illustrated in Fig. 5h-i and follows a growing non-linear pattern. To generate the following result, the simulation run multiple times for each muscle strength position while increasing the number of parallel CmCs until a low intention tremor threshold was consistently reached. The threshold was set to the average intention tremor observed in the Fig. 5d, representing a very smooth continuous movement. Muscle strength was defined as the maximum force that a muscle can exert on the joint in 1 time-sample (Kell et al., 2001). This measure corelates with the number of functioning muscle fibers within the muscle unit, as a loss of muscle units often leads to a decrease in muscle strength (McNeil et al., 2005). The left-hand columns of Fig. 5h-i show the number of CmC’s required to maintain a low intention tremor in the movement. The magenta dashed line represents a fit of the data using a 6 th order polynomial. The results are expressed using both linear and logarithmic scales to highlight the results for low muscle strength results. These graphs indicate that as muscle strength increases, the number of CmC’s assigned to the muscle unit must simultaneously increase in order to accommodate and perform a smooth movement. This relationship is increasingly non-linear, meaning that an increase in muscle strength requires a larger non-proportional increase in the number of CmCs running in parallel. Regarding the clinical symptoms of neocerebellar syndrome, it is known that intention tremor disproportionally affects the proximal muscles which exert more strength (Bodranghien et al., 2016).

#### Acute Alcohol Intoxication and the Cerebellum

**Fig. 6:**
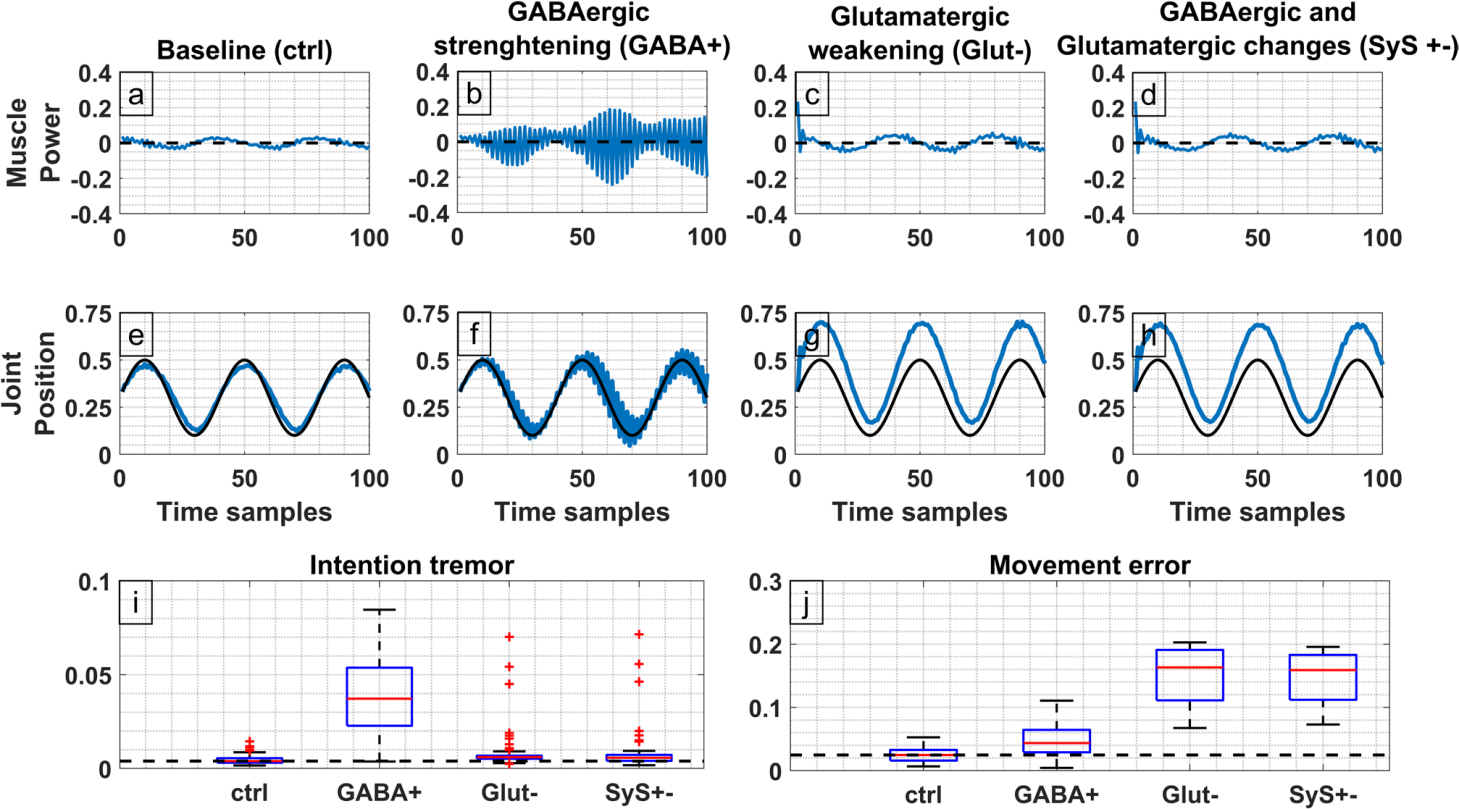
Simulated effects of ethanol on cerebellar micro complexes and movement dynamics. Based on literature ethanol is expected to facilitate GABA and inhibit Glutamate neurotransmission. **a**-**d**) The blue line represents muscle power against time. The dashed black line shows zero power. **e**-**j**) The blue line shows the actual joint position. Black line shows the intended joint position. **i**) Boxplot of measured intention tremor (Section "Intention tremor", Eq. 12) for different simulated changes in synaptic weights. **j**) Boxplot of movement error (Section "Muscle movement model rationale" Eq. 22) for different simulated synaptic weights

Acute alcohol intoxication has been long known to cause neocerebellar like symptoms mainly comprised of ataxia and dysarthria (Setta et al., 1998). The exact mechanism that causes neocerebellar like symptoms in the acute phase of alcohol intoxication remains unknown although long-term alcohol consumption has been linked with cerebellar degeneration (Mitoma et al., 2021). This effect is likely not present in acute alcohol intoxication as physiological function is restored after ethanol is effectively cleared from the bloodstream. Albeit the exact effect exerted on the electrical activity of the CmC neurons by ethanol remains unknown, it is generally accepted that ethanol allosterically binds and activates GABAa receptors (Davies, 2003) while also exerting a weaker inhibition of glutaminergic synapses (Tsai & Coyle, 1998). To model the observed effects of ethanol in CmC neurons, the SyS (Section "CmC model implementation using histologic Boolean mapping") of all GABAergic synapses was increased and the SyS of all glutaminergic synapses was decreased (to a lesser extent). The results of this simulation are presented in Fig. 6. The first row shows the muscle power (force) exerted to the joint using a blue line (Fig. 6a-d). The second row shows the achieved joint position with a blue line and the desired position with a black line (Fig. 6e-h). The aforementioned rows are separated into 4 columns: first column being the baseline CmCs (unaltered SyS), second column shows the same circuit under identical conditions but after increasing the SyS for all GABAergic synapses. Similarly, the third column shows the model after decreasing the SyS for all Glutaminergic synapses. Finally, the fourth column shows the combined effect of the GABAergic and Glutaminergic modifications previously described. The intention tremor (Section "Intention tremor", Eq. 12) and error against the desired position (Section "Muscle movement model rationale", Eq. 22) are shown in the boxplots of the bottom row of Fig. 6i-j) for each respective synaptic manipulation. These results, suggest that by increasing the strength of GABAergic neurotransmitters, a significant margin of tremor and error emerge in the achieved movement similarly to the expected neocerebellar like symptoms that arise from acute alcohol intoxication. A decrease in glutamatergic synapses significantly increases the error but nevertheless does not give rise to the same magnitude of tremor seen in the GABAergic positive modulation. By combining both the GABAergic and glutamatergic modifications an intermediate state is reached with significant increases in both tremor and error compared to baseline, but nevertheless, decreased when compared with the isolated modifications. This suggests a partial compensation of intention tremor, not observed for the movement error (dysmetria). In summary, this simulation predicts two main cerebellar symptoms (intention tremor and dysmetria) observed during the acute phase of alcohol intoxication.

### CmC Adaptive and Learning Behaviour

#### Cerebellar Compensation for External Forces

**Fig. 7:**
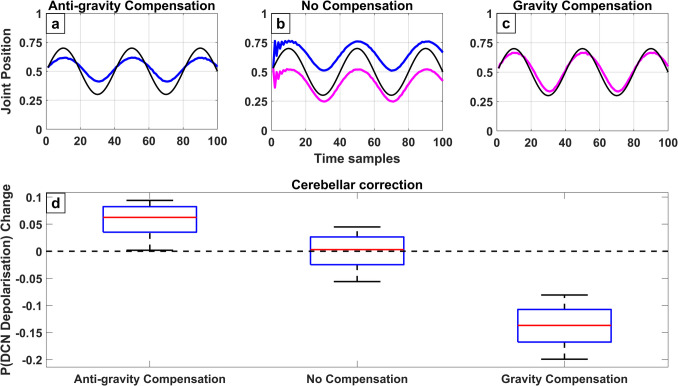
Estimated DCN excitability changes for adaptation to external forces affecting the joint. Gravitational force pushes joint position towards extension (value of 0) whereas anti-gravitational force pulls the joint towards flexion (value of 1). **a**-**c**) The blue line indicates the position of a joint affected with an anti-gravitational force and the magenta line for a joint affected by a gravitational force. The black line shows the desired joint position. **d**) Boxplot of the change of DCN firing probability in each type of compensation. Dashed black line shows no change in firing probability

In our daily lives, our body and limbs are exposed to either assisting or opposing forces affecting the desired motor plan. The balance of muscle power exerted on a joint by the agonist and antagonist muscles require a fine control from the central nervous system. The adaptive role of the cerebellum regarding external forces has long been theorised and observed in the literature (Richter et al., 2004; Roth et al., 2013). The question that this study asks, is what role the cerebellum plays in correcting the executed motor plan when external forces are introduced. As discussed, in Section "Muscle movement model rationale" and "CmC HBM-VNR function summary" this model is inspired by the cerebellar brain inhibition and relies on the finding that a tonic inhibition of the DCN by the Purkinje cells assists the desired movement by selectively inhibiting the antagonist muscles. As a result, theoretically when a force opposing motion as gravity is introduced, the DCN should be modified to exert a decreased activity and vice versa to exert an increased activity when a force assisting the desired movement is present. Figure 7b illustrates how movement would change if the default circuit was to act when gravity (pink line) and antigravity (blue line) forces are introduced. Thus, when a force is applied to the default circuit it causes the movement to over and undershoot respectively. The model was then modified according to the expected cerebellar response to achieve compensation for the external forces. Figure 7c shows how the cerebellum can compensate for gravity to achieve the desired movement by reducing the probability of the DCN firing through a tonic inhibition Fig. 7d. On the other hand, panel a illustrates how a tonic disinhibition on the DCN can partially compensate for a force assisting movement. The model shows that the cerebellar compensation for forces assisting motion seem to be less effective compared to compensation for forces opposing motion. Regardless, the effects of the DCN modifications in the model, suggests a possible mechanism for the adaptive role of the cerebellum to external forces, without introducing any structural changes on the CmC.

#### Synaptic Plasticity and the Adaptive Cerebellar Model

**Fig. 8:**
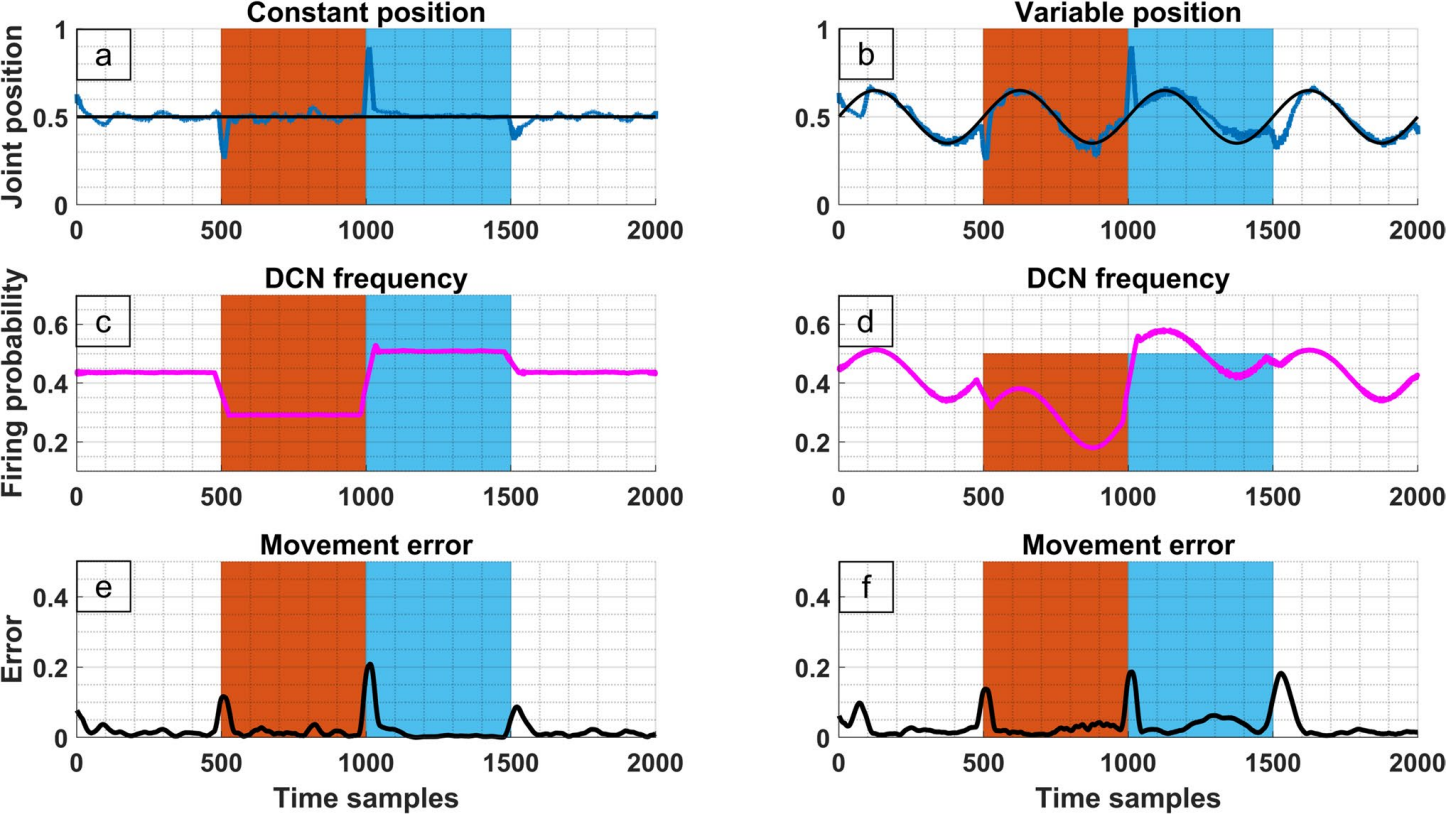
Cerebellum adaptive model undergoing real-time synaptic modifications in order to adapt to external forces. Gravitational force (red superimposed background) pushes joint position towards extension (value of 0) whereas anti-gravitational force (blue superimposed background) pulls the joint towards flexion (value of 1). Frequency is expressed in the model equivalent units of firing probability (Section "CmC model implementation using histologic Boolean mapping", Eq. 4). Figure 8**a**-**b**) The blue indicates muscle power against time and the black line shows zero power. **c**-**d**) The magenta line shows the DCN firing frequency against time. **e**–**f**) The black line shows the movement error (Section "Muscle movement model rationale", Eq. 22) across time

Activity dependent plastic changes have been described in several synapsis of the CmC that emerge without noticeable changes in the cytoarchitectural structure. These synapse modifications fall within two main categories: Long-Term Depression (LTD) and Long-Term Potentiation (LTP) which, respectively diminish and enhance the synaptic strength. Table 2 shows a summary of the activity dependent synaptic modifications that were implemented in the model following literature reports. They were incorporated into the cerebellar model by increasing or decreasing the SyS variable for LTP and LTD respectively. Figure 8 illustrates how the adaptive model incorporating the modifications seen in Table 2 behaved with a changing load applied to the joint. The top row (Fig. 8a-b) shows the desired position with a black line, and the actual position with a blue line. The middle row (Fig. 8c-d) shows the mean firing probability of all the CmCs. The bottom row (Fig. 8e-f) shows the error defined as the difference between the actual and desired positions (Section "Muscle movement model rationale", Eq. 22). Red and blue backgrounds in Fig. 8 indicate the presence of a force applied to the joint, red for gravitational force (pushing joint position towards 0) and blue for antigravitational force (pushing towards 1). None colored background was used when no external forces applied to the joint.

By implementing the literature reported plastic changes, the adaptive model was able to dynamically accommodate to a changing kinetic environment, which was simulated by changing the forces applied on the joint. During the onset and offset of the kinetic change (beginning and end of the superimposed coloured background, Fig. 8) a large spike in the measured error was observed. However, within few time samples of the simulation the error returns to its baseline value, indicating that the synaptic plasticity changes have achieved a successful adaptation. The same effect was observed in the top row, where the matching between of the black and blue lines was restored following a transient deviation. The middle row describes how the collective synaptic modifications affect the DCN’s output frequency. During a downward force (red background) a downward shift of the DCN’s frequency was observed, and vice versa an upward force (blue background) caused an increased DCN frequency. Nevertheless, removal of the forces, caused the DCN frequency to return to the same baseline frequency observed before the perturbations were applied. Importantly, the observed changes in the DCN frequency of the adaptive model matched the theorised changes that are described in Section "Cerebellar compensation for external forces" and Fig. 7. Furthermore, the implemented modifications still allow the joint to pursuit both constant and variable desired positions.

## Discussion

### Comparison With Biological Observations

#### Single CmC Firing Pattern During Movement

Recordings of the cerebellum show increased activity during ipsilateral movement whether the movement corresponds to flexion or extension. This effect arises as separate CmCs are assigned to both flexor and extensor muscles and are measured during these studies (Dorigo et al., 2023; Sauerbrei et al., 2015). On the other hand, when a single CmC is measured during flexion to extension cycles a cyclical pattern is observed, involving a higher frequency during one phase of the movement followed by reduced spike frequency on the opposite phase (Frens et al., 2001). The same pattern was generated by the HBM-VNR model of a single CmC during movement (Section "Single cerebellar circuit across movement" and Fig. 2). Additionally, the model shows variability between the repetitions of movement which is also observed in real-life recordings (“Neuroscience, 5 th Ed.,” 2012). Therefore, to remove variability and obtain a pattern of DCN activity driven by both afferent sources, the decision was taken to simulate and average multiple CmCs in parallel. Another strength of the HBM-VNR model is that it computes and reports the simultaneous activity of all neurons within the CmC, which would be experimentally very challenging. The HBM-VNR approach further provides simplicity as the Boolean algebra expressions can be easily run with low processing power compared to previous modelling approaches using differential equations (Hodgkin & Huxley, 1952). Concurrently, synaptic plasticity rules can be implemented in the HBM-VNR model (Section "Synaptic plasticity and the adaptive cerebellar model", Fig. 8). Thus, the model allows us to study the effect of CmC population size as well as how synaptic plasticity can affect movement, highlighting that the function of a brain region must be interpreted in terms of circuit interactions.

#### CmC Firing Frequency Changes During Channelopathies

HBM-VNR can further be used to model the changes in firing patterns and frequency of neurons within the CmC according to specific channelopathies. A similar phenomenon was reproduced by Solouki et al. (2022) who utilised a firing rate model to show how optokinetic reflex impairments can be mapped onto specific synapse level synaptic dysfunctions. This was explored in Section "KCNA1 mutation" and "CACNA1 A rocker and tottering mutations" as a form to validate the biological modelling functionality of the HBM-VNR approach using mutations with known changes to the Purkinje cells firing properties. Section "KCNA1 mutation" describes the observed changes for the KCNA1 mutation that causes a loss of function channelopathy on the Kv1.1 channel, whereas Section "CACNA1 A rocker and tottering mutations" explores the effects of the tottering and rocker mutations of the CACNA1A gene. The Kv1.1 channelopathy which is associated with episodic ataxia type 1 (EA1), was simulated by lowering the DT of inhibitory interneurons. This led to enhanced inhibition of Purkinje cells and a subsequent reduction in their firing frequency and regularity, resembling the silencing and burst disruption observed in Kv1.1-null mice (Martin & Kullmann, 2023; Zhang et al., 1999). Although, not explicitly measured by studies, HBM-VNR revealed a subsequent episodic high-frequency firing of the DCN cells that could theoretically correspond to the motor overactivity seen in EA1. On the other hand, CACNA1A mutations were modelled by altering the SyS of affected neurons to model the reduced neurotransmitter release due to impaired calcium influx. Two degrees of severity were explored, the tottering variant (65% reduction) and the rocker variant (85% reduction). These changes reproduced the expected burst-pause pattern in Purkinje cell and erratic DCN activations in increasing severity from the tottering to rocker variants, as reported in the literature (Cook et al., 2021; Stahl & Thumser, 2014). In addition, HBM-VNR was able to simulate the firing pattern of the downstream Golgi and inhibitory interneurons that may explain the firing pattern observed in the DCN and Purkinje cells. Simulating the simultaneous firing activity of all the neurons within the CmC offers a systems-level explanation for the progressive motor phenotype that is experimentally difficult to measure in real-life studies. This highlights HBM-VNR’s framework ability to explore the effect of circuit specific changes.

#### Simulation of Neocerebellar Syndrome and Acute Alcohol Effects

Intention tremor refers to the impaired modulation of movement due to dysfunctional cerebellar output pathways, particularly involving the dentate nucleus and its connections to the thalamus and motor cortex. This is attributed to the defective feedback control mechanisms, which lead to increased, uncoordinated activity when attempting goal-directed actions (Kakei et al., 2021). The HBM-VNR model successfully mimics intention tremor by decreasing the number of functioning CmC (Section "Neocerebellar syndrome" and Fig. 5a-g). In addition, HBM-VNR conveys that as the muscle power increases, the number of CmCs required to coordinate movement and avoid intention tremor increases as well (Section "Relationship of intention tremor and number cerebellar circuits" and Fig. 5h-i). Figure 5 illustrates the increasing non-linear relationship between muscle power and number of CmC required for a smooth movement. Existing literature suggests that proximal muscles are more affected by intention tremor in neocerebellar syndrome (Bodranghien et al., 2016). This is in accordance with our findings in the model simulation, since proximal muscles have a higher power output, thus requiring a greater number of CmC to overcome intention tremor. Therefore, if a topographically uniform loss of CmCs within the cerebellum occurs, it is more likely that the proximal (stronger) muscles would be disproportionally affected as they require an increasing number of CmCs to perform smooth movement. The model is further able to simulate and suggest how cerebellar syndrome like symptoms arise in the acute phase of alcohol intoxication. This was achieved by emulating the reported GABA and Glutamate synaptic effects of alcohol (ethanol) (Section "Acute alcohol intoxication and the cerebellum" and Fig. 6). Furthermore, in accordance with the CBI theory, electrical stimulation of the cerebellum causes a reduced muscle evoked potential. This experimental fact is represented in the HBM-VNR model as a decrease in the muscle power as the number of CmC increases (Sections "CmC HBM-VNR movement simulations" and Figs. 5 and 6).

#### Motor Adaptation to External Forces

In Section "Synaptic plasticity and the adaptive cerebellar model", the adaptive cerebellar model is described which incorporates the observed plastic changes (Table 2) from the literature into the set of Boolean equations used to implement the HBM-VNR model described in Section "CmC model implementation using histologic Boolean mapping". If the conditions for synaptic plasticity are not met for a certain period, the synapse strength returns to its initial (baseline) value. The significance of the adaptive model is that it can more closely follow the desired movement compared to the pure set of Boolean algebra equations alone and achieves increasing movement accuracy over time. After a few simulation time samples, the adaptive model’s individual synaptic strength (of each neuron synapse) stabilises and results in a steady state, that follows the desired movement very closely, while also retaining the ability to accommodate to a new set of movements (Section "Synaptic plasticity and the adaptive cerebellar model", Fig. 8). The VNR model implemented here accounts for a variable synaptic depolarisation following the release of neurotransmitter in contrast to the deterministic differential equation models (Izhikevich, 2001; Köhn & Wörgötter, 1998). The strategy implemented in this study relies on the firing probability without an explicit simulation of the membrane potential (Section "Relationship between firing probability and firing frequency", Eqs. 4 and 5). The HBM-VNR approach can account for the response variability of the different units, producing a continuous output in the frequency spectrum driven by the afferents. Thus, the HBM-VNR model allows for complex behaviour to arise through the interaction of basic neurons within neural networks of realistic architecture. HBM-VNR requires multiple neural networks to run in parallel for a consistent movement pattern to arise, reflecting the repetitive crystal-like structure of the cerebellum. In summary, the results of this study explain why multiple repeating circuits are required to perform smooth movements (Sections "Neocerebellar syndrome" - "Relationship of intention tremor and number cerebellar circuits") using a repeating stereotyped cytoarchitecture, as observed in the three physiological divisions of the cerebellum (vestibulo-, spino- and cerebro-cerebellum).

#### Variability in Physiological Recordings

To the EEG and MEG community it is often known that an identical stimulus (evoked response) often yields a different electrical activity within the brain. This effect is true for visual (Heggelund & Albus, 1978), auditory (Thomas et al., 1989) and somatosensory stimuli (Koutras et al., 2008) as well as for responses to perceived errors such as in working memory tasks (Nakuci et al., 2023). When comparing independent STs from the same stimulus, even within the same subject, large variations are observed. However, these are largely masked due to the averaging of multiple STs that is a common practise in EEG and MEG studies. Although this approach results in a smooth “appealing” waveform that is similar between subjects and increases the signal-to-noise ratio, it removes high-frequency components while preserving only the common low-frequency components. Nevertheless, a recent analysis of somatosensory responses revealed that two different networks can be activated from the same somatosensory stimulus (Karittevlis et al., 2023). This variability has long been observed, with some studies explaining its emergence due by variations in the cortical excitability (Arieli et al., 1996; Kenet et al., 2003), while other studies suggested that the variability could be an intrinsic feature of the system (Ioannides et al., 2002). The observed large-scale variability would not be able to occur in the context of a deterministic model. This study provides an explanation for the observed variability through the randomisation of the neuronal responses in the VNR model. The idea that varied plasticity is required for motor movement is also supported in multiple models both utilizing spike neurons (Antonietti et al., 2016) and in closed-loop robotic simulations (Garrido et al., 2013).

### Biological Insights

#### SCFH and the Comparator Functionality of the CmC

Section "Population of CmC behaviour" describes the SCFH, explaining the mechanism by which the CmC exerts its comparison functionality (D’Angelo & Casali, 2013; West & Gelderd, 2003). The comparator role of the CmC becomes apparent when considering the DCN and climbing fiber frequencies (efferent and afferent respectively). Climbing fibers set the DCN’s central output frequency which is then symmetrically modulated by the difference between the climbing fiber and mossy fiber frequencies, resulting in equal but opposite shifts in the DCN frequency. In other words, an upward shift is caused if mossy fiber frequency is greater than the climbing fiber frequency and vice versa. An inference that can be made from Section "Population of CmC behaviour" is that motor learning information is stored within the individual CmCs in the form of plastic changes. This can be deduced by comparing the columns of Fig. 4d-f, where the line of symmetry (central frequency) is translated due to specific synaptic alterations. This view is consistent with classic theories identifying the CmC as the basic adaptive unit of cerebellar learning (Albus, 1971; Ito, 1984; Marr, 1969) who theorised that the CmC is the basic learning unit where motor memory is encoded as synaptic changes. Therefore, these localized plastic changes allow the cerebellum to store motor learning in modular, context-specific units. In summary, SCFH’s perspective aligns with the previous internal model theories (D’Angelo & Casali, 2013; Ito, 2006) while also providing a mechanistic explanation grounded on the CmC’s histology.

#### Model Findings and Previous Cerebellar Hypotheses

The HBM-VNR framework presented in this study offers a neural circuit modelling method by translating the histological architecture directly into Boolean algebraic equations. The procedure was utilised to create a motor control model based on the CmC. This approach differs from traditional cerebellar models that rely on differential equations or spiking neural networks, such as the Cerebellar Model Articulation Controller (CMAC) (Albus, 1971) and Feedback-Error Learning (FEL) models (Kawato et al., 1987). The HBM-VNR model of the cerebellum agrees with the earlier models in that they all establish the role of the cerebellum as an adaptive feedforward controller. However, HBM-VNR offers the advantages of being grounded on the anatomical details and has relatively low computational demands. More biologically detailed spiking models (Carrillo et al., 2008; Garrido et al., 2013) replicate granule cell layer dynamics and plasticity, but face similar scalability challenges. HBM-VNR addresses this problem by utilising simple, and efficient Boolean equations based on realistic circuital cytoarchitecture and modelling multiple parallel CmCs to cancel variability. Concurrently, the modularity of the HBM-VNR framework allows for the incorporation of biologically observed plasticity mechanisms (Table 2). This enables replication of complex motor behaviours such as intention tremor from CmC loss, adaptive responses to external forces, and ethanol-induced motor deficits. Notably, HBM-VNR provides quantitative insights into the non-linear relationship between muscle strength and required CmC numbers, shedding light on why proximal muscles are more affected in cerebellar disorders—a finding aligned with clinical observations (Bodranghien et al., 2016). Furthermore, HBM-VNR captures cerebellar brain inhibition effects and adaptive motor control, aligning with Smith Predictor and adaptive filter models (Garrido et al., 2013; Miall et al., 1993). Given HBM-VNR’s light computational requirements, inherent modularity and demonstrated success in modelling the CmC, HBM-VNR is suggested as a potential modelling framework for applying similar methodologies in other brain regions, as more detailed histological maps become available.

### Technical Insights

#### Comparison with Spiking Models

Classical spiking neuron models, such as the HH model (Gerstner et al., 2014; Hodgkin & Huxley, 1952) and Izhikevich (Izhikevich, 2003) describe the evolution of membrane potential through coupled differential equations. These models can account for the spiking behaviour, refractory period, and threshold property as part of their intrinsic dynamics. In other words, these properties do not need to be artificially introduced, as in the case of the leaky integrator and fire neurons. For example, in HH and other ionic current based models, the threshold effect is created by a cascade of openings of voltage dependent sodium channels, and the refractory period by a combination of sodium channel inactivation and potassium channel opening. However, these models come at a high computational cost when scaled to simulate network-level phenomena. Different levels of reductionism can be applied preserving the basic input output relations to decrease the computational burden. In this context, the HBM-VNR model does not represent the voltage as an explicit variable but rather focuses on the firing pattern generated in response to afferent signals. This is achieved through a binary modelling of neuronal output created by a simple numerical representation of synaptic dynamics and threshold properties. In each time sample of the simulation a neuron can only have an output of 0 or 1, which is fed to the following neurons. This binary value, defined by the equations in Section "CmC model implementation using histologic Boolean mapping", is the result of the input received and a random number dawn from a uniform distribution independently generated for each pre to post synaptic interaction.

The idea behind the HBM-VNR approach stems from the field of artificial neural networks, where neurons receive input signals and process them using weights to produce an output signal. Neurons are organized into layers, with information flowing from the input layer through hidden layers to the output layer. This concept was implemented in a cerebellar model by Solouki et al. (2019) who introduced the term transmission coefficient to model synaptic interactions. Similarly to this approach, HBM-VNR utilised SyS to quantify the effect that each neuron type exerts on the next neurons of the histologically inspired circuit. Thus, instead of an organization in sequential layers, the transfer of information is determined by the known cytoarchitectonic structure. The collective effect of all synaptic weights can be visualised in the SCFH, which explains how the DCN frequency shifts based on the afferent input frequencies and collective SyS and DT across the parallel circuits. The main advantage of our reductionist approach is a substantial decrease in computational burden, achieved by the replacement of the differential equations by logical operations, including a randomization factor and parallel processing for each CMC unit. The voltage is not modelled as a variable, but the input output relations measured. The input–output relation is represented by the so-called gain function. An example of this gain function is provided in Fig. 4a, b, c. The DCN frequency is given by the colour map dependence on the afferent mossy fibber and climbing fibre frequencies. Our model produces a roughly linear input–output relation with saturation. The saturation is reached at lower input firing probability (frequency) for larger SyS values. In the HH model (Gerstner et al., 2014), this function is usually linear with saturation for high input values. Thus, our model can produce a realistic gain function, like the one of HH, with a very simple and computationally efficient algorithm. In addition, the synaptic rules capture the basic features of LPT and LTD (SyS) as described in each synapsis of the cerebellar circuit, and the effects of neuromodulators (DT).

#### Insights to EEG and MEG Studies

SCFH highlights the importance of contextualizing output frequencies relative to baseline, offering evidence toward the relevance of interpreting neural oscillations in EEG and MEG studies against their ground state (Ioannides et al., 2017). This emphasizes the importance of considering the frequency of surrounding neural networks when studying a specific area. Comparing the network’s activity to its ground state prior to the event of interest can offer insight into whether the change reflects inhibition or excitation. In MEG and EEG studies, low delta frequencies (1–4 Hz) are often associated with inhibition (Harmony, 2013), while gamma frequencies (30–100 Hz) are linked to activation (Kucewicz et al., 2017). However, this general rule may oversimplify complex dynamics, especially during human sleep. Sleep is divided into 5 stages; eyes-closed wakefulness (ECW), NREM1, NREM2, NREM3, REM, and we may also consider the putative REM0 sleep stage (Ioannides et al., 2022; Orphanides et al., 2024). Each stage exhibits characteristic cortical activations, such as vertex sharp waves in NREM1 and K-complexes or spindles in NREM2. Although these features occur in low-frequency bands typically associated with inhibition, they are considered excitatory because their frequency is elevated relative to the stage’s baseline. Thalamocortical excitability changes across sleep stages due to varying neuromodulator levels from the brainstem (Scammell et al., 2017; Siegel, 2004). Therefore, establishing a baseline from quiet periods within each sleep stage is critical. By comparing the frequency spectrum between the large graphoelements (GLEABS) and the background activity of the corresponding sleep stages, a 2022 study was able to identify the Nucleus Basalis Meynert as their common generator (Ioannides et al., 2022). Within this context, the SCFH supports this strategy, emphasizing the importance of comparing transient frequency changes relative to their background activity.

#### Factors Impacting Motor Control

Several factors can affect joint movement such as muscle fatigue (Enoka & Duchateau, 2007) and enhanced friction due to joint degeneration (e.g., Osteoarthritis, (Felson, 2006)). Some of these factors could be implemented into the model by changing the parameters shown in Eqs. 20–21. It is possible to incorporate muscle fatigue by applying a time-dependent reduction variable with an initial value of 1, which then can decrease towards 0 as the simulation runs. If this variable is multiplied by the “Muscle Strength” parameter (Eq. 20) we can obtain a simulation of muscle fatigue. In the scope of the model, the time-dependent reduction of muscle power could be the equivalent of muscle fatigue due to progressive loss of contracting muscle fibres, i.e., exercise induced neuromuscular fatigue (Taylor et al., 2000). Enhanced friction due to osteoarthritis could also be implemented by multiplying “Muscle Power” (Eq. 20) by a constant variable ranging from 1 to 0. This would introduce a fractional reduction that could emulate the constant force of friction. Muscle imbalance could also be introduced to the model by implementing different weights in the “extensor” and “flexor” variables in Eq. 20, which would correspond to either the extensor or flexor muscle being able to generate more force in each time-sample. However, there are other factors such as the effect of age (Vandervoort, 2002) and axon demyelination (Waxman, 1980) on axonal conduction speed that, although are very important in muscle control cannot currently be simulated using the current HBM-VNR model. Incorporating the physiological parameters discussed above into the model can increase the simulating capabilities in future versions.

#### Applications and Future Work

In recent years there has been a significant improvement in accurate high-resolution neuronal mapping. These maps can specify the position of neurons and synapses in, for example, insect larvae (Winding et al., 2023), house flies (Dorkenwald et al., 2024; Schlegel et al., 2024) and of the human temporal cortex (Shapson-Coe et al., 2024). In this context, the VNR model and HBM-VNR approach presented in this study, could be implemented into these maps to supplement existing evidence, or to disprove the validity of the HBM-VNR method for a specific large scale neural network system. These applications might serve as a tool to understand the mechanisms underlying the functions of the cytoarchitectonic areas mapped. HBM-VNR can model and monitor the activity of each neuron within a circuit and simultaneously provide insights on network interactions. Serendipitously, this work has also resulted in the creation of a biologically inspired neural network, that follows a desired movement while adapting to external and internal forces. This network could theoretically be implemented in a motion control system using either conventional digital computers or even analogue computers, as was the case during the pioneering stages of neural network theory development (Rosenblatt, 1958). The HBM-VNR model could also be used to predict the effects on neuronal firing and circuit behaviour following the introduction of a pharmacological agent or channelopathy as was showcased in Sections "KCNA1 mutation" and "CACNA1 A rocker and tottering mutations".

### Limitations

#### Simplification of Biological Circuits

The histologically inspired circuital architecture is limited to the cerebellum, omitting crucial contributors to motor control, such as the motor cortex, brainstem, and spinal feedback loops. The effects of these neural networks are included in a highly reductionist manner, through two inputs to the cerebellar circuit coming from the motor cortex, and spinal cord, representing the target and actual positions of the joints. While this is justified to focus on the error-minimizing properties of the cerebellar parallel units, further work is needed to include a more comprehensive representation of the motor system. In addition, the findings need to be reproduced with detailed ionic based neuron models, capturing the oscillatory properties often displayed by real neurons, even in response to constant inputs. These membrane oscillations (driven for example by calcium currents, persistent sodium and potassium currents) can underlie busting firing and were not implemented in our model and might bear important consequences in motor control. Even missing the simplicity and low computational burden of our simulations, the effects of these circuital and intrinsic cellular properties are worth to be investigated. Although the HBM-VNR model can simulate the motor correction of the basic cerebellar units, we need to consider the full motor system to allow more complex behaviours to emerge. These interactions will be explored in future work.

#### Boolean Simplification of Synapses

Despite the approach proposed in this study there are some limitations and sources of bias that need to be acknowledged. One of the main shortcomings is the simplification of neurons to an either on or off state in each time sample due to the usage Boolean algebra and so the membrane potential is not explicitly simulated. Further, temporal summation is not accounted for. In this context, the argument can be made that the combination of large circuits running in parallel with built-in variability can collectively represent subthreshold states through the mean firing probability. Moreover, the integrated randomisation of the neurons replicates the natural variability observed in physiological recordings, although grossly simplifying the underlying biological mechanisms. For example, details related to synaptic function (Stange-Marten et al., 2017) and neuromodulatory interactions (Marder, 2012) that underline the observed spiking variability were not accounted for. Additionally, the present simulations only account for one of the functions of the cerebellum, which is movement, not accounting for others such as cognition, and emotional behaviour (Fastenrath et al., 2022). However, the argument could be made that these complex behaviours arise from the interactions of the cerebellum with other brain regions (e.g., limbic system) which is a direction for future work but is not within the scope of this study.

### Conclusion

This study introduces HBM-VNR as an anatomically inspired computational framework that directly translates neural histological maps into functional Boolean logic models while preserving realistic circuital connectivity. HBM-VNR is based on a synaptic model “VNR” proposed in this study and the concept of modelling frequency as firing probability to allow for the successful representation of synapses as Boolean expressions. HBM-VNR allows for a model that is computationally efficient and versatile, while also being able to incorporate synaptic plasticity rules. By simulating individual cerebellar micro complexes and integrating the VNR synaptic model to reflect physiological variability, the model replicates essential cerebellar functions including similar patterns of Purkinje and DCN firing as that reported in the literature, adaptive motor control, compensation for external forces, and the emergence of pathological conditions such as intention tremor and ethanol motor effects. Additionally, the SCFH offers a biologically plausible mechanistic explanation for the cerebellar comparator function, aligning with the internal model theories. SCFH provides evidence towards the classic view that the cerebellar units are the basis of motor learning encoded as synaptic modifications. In conclusion, HBM-VNR bridges histological structure and functional modelling creating a scalable framework and methodology that can be extended to other brain regions as large-scale histological datasets become available (Table 3).

**Table 3:** Abbreviations used in the manuscript

Abbreviations	
Deep Cerebellar Nucleus	DCN
Purkinje cell	PC
Climbing Fiber	CF
Cerebellar Brain Inhibition	CBI
Mossy Fiber	MF
Shifting Central Frequency Hypothesis	SCFH
Single Trial	ST
Electroencephalography	EEG
Magnetoencephalography	MEG
Cerebellar Circuit	CC
Cerebellar micro complex	CmC
DT	Depolarization Threshold
SyS	Synapse Strength
Excitatory post-synaptic potential	EPSP
Inhibitory post-synaptic potential	IPSP
Histologic Boolean Mapping	HBM
Variable Neuronal Response	VNR
Episodic Ataxia 1	EA1

## Information Sharing Statement

No dataset were generated or analysed during the current study as this study is purely computational. The HBM-VNR cerebellum model is publicly available in the form of a MATLAB function alongside a simple MATLAB script example which can be found in the following GitHub repository: https://github.com/gregorph/HBMVNR.

## Data Availability

No datasets were generated or analysed during the current study.
